# Effect of IL-17A on the immune response to pulmonary tuberculosis induced by high- and low-virulence strains of *Mycobacterium bovis*

**DOI:** 10.1371/journal.pone.0307307

**Published:** 2024-07-18

**Authors:** Yadira Rodríguez-Míguez, Vasti Lozano-Ordaz, Angel E. Ortiz-Cabrera, Jorge Barrios-Payan, Dulce Mata-Espinosa, Sara Huerta-Yepez, Guillermina Baay-Guzman, Rogelio Hernández-Pando

**Affiliations:** 1 Programa de Doctorado en Ciencias Biomédicas, Universidad Nacional Autónoma de México, Mexico City, Mexico; 2 Departamento de Patología, Sección de Patología Experimental, Instituto Nacional de Ciencias Médicas y Nutrición “Salvador Zubirán”, Mexico City, Mexico; 3 Unidad de Investigación en Enfermedades Oncológicas, Hospital Infantil de México “Federico Gómez”, Mexico City, Mexico; Rutgers Biomedical and Health Sciences, UNITED STATES OF AMERICA

## Abstract

Tuberculosis (TB) is an infectious, chronic, and progressive disease occurring globally. Human TB is caused mainly by *Mycobacterium tuberculosis* (*M*. *tuberculosis*), while the main causative agent of bovine TB is *Mycobacterium bovis* (*M*. *bovis*). The latter is one of the most important cattle pathogens and is considered the main cause of zoonotic TB worldwide. The mechanisms responsible for tissue damage (necrosis) during post-primary TB remain elusive. Recently, IL-17A was reported to be important for protection against *M*. *tuberculosis* infection, but it is also related to the production of an intense inflammatory response associated with necrosis. We used two *M*. *bovis* isolates with different levels of virulence and high IL-17A production to study this important cytokine’s contrasting functions in a BALB/c mouse model of pulmonary TB. In the first part of the study, the gene expression kinetics and cellular sources of IL-17A were determined by real time PCR and immunohistochemistry respectively. Non-infected lungs showed low production of IL-17A, particularly by the bronchial epithelium, while lungs infected with the low-virulence 534 strain showed high IL-17A expression on Day 3 post-infection, followed by a decrease in expression in the early stage of the infection and another increase during late infection, on Day 60, when very low bacillary burdens were found. In contrast, infection with the highly virulent strain 04–303 induced a peak of IL-17A expression on Day 14 of infection, 1 week before extensive pulmonary necrosis was seen, being lymphocytes and macrophages the most important sources. In the second part of the study, the contribution of IL-17A to immune protection and pulmonary necrosis was evaluated by suppressing IL-17A via the administration of specific blocking antibodies. Infection with *M*. *bovis* strain 534 and treatment with IL-17A neutralizing antibodies did not affect mouse survival but produced a significant increase in bacillary load and a non-significant decrease in inflammatory infiltrate and granuloma area. In contrast, mice infected with the highly virulent 04–303 strain and treated with IL-17A blocking antibodies showed a significant decrease in survival, an increase in bacillary loads on Day 24 post-infection, and significantly more and earlier necrosis. Our results suggest that high expression of IL-17A is more related to protection than necrosis in a mouse model of pulmonary TB induced by *M*. *bovis* strains.

## Introduction

Tuberculosis (TB) is a chronic bacterial disease caused by *Mycobacterium tuberculosis* (*M*. *tuberculosis*) complex species. *M*. *tuberculosis* is the main causative agent of human TB [1]. About a fourth of the world’s human population is latently infected with *M*. *tuberculosis*; however, only 5% will experience reactivation and suffer progressive disease. In 2022, the World Health Organization reported an estimated global total of 10.6 million people with active TB and 1.3 million deaths, making TB the second leading infectious killer after COVID-19 [2]. Another member of *M*. *tuberculosis* complex is *Mycobacterium bovis* (*M*. *bovis*). Cattle are the primary hosts of *M*. *bovis*, and due to the close interaction between cattle and humans, *M*. *bovis* is a zoonotic risk [3, 4]. Limited disease management and the uncontrolled movement of infected cattle are factors that contribute to the ongoing transmission of *M*. *bovis* [1, 5, 6]. Zoonotic transmission of this pathogen to humans occurs primarily through close contact with infected cattle or the consumption of contaminated animal products, such as unpasteurized milk or contaminated dairy animal products [7–9], leading mainly to extrapulmonary manifestations of human TB [3, 10]. Zoonotic TB is still widespread worldwide, with low- and lower-middle-income countries historically having a higher burden of *M*. *bovis* infections [11].

TB is a chronic infection that produces significant immunological abnormalities, which influence the rate of disease progression, the extension of inflammation, the bacterial load and replication [12, 13], as well as the production of different types of lesions, such as granulomas, necrosis, or fibrosis that affect the lung tissue in diverse proportions. Necrosis has been defined as unprogrammed cell death, histologically characterized by cell swelling, with eventual breakage of the cell membrane that produces an inflammatory response due to the leakage of cellular contents, representing the spectrum of cellular death changes in tissue due, in part, to the progressive activity of released hydrolytic enzymes [14]. TB caused by *M*. *bovis* mainly affects the lungs and lymph nodes and is characterized by the formation of caseous and necrotizing granulomas [15], which are a consequence of the interaction between the immune response and the pathogen’s virulence factors [16].

To study the interaction between the immune response and mycobacteria in the lungs, several animal models are used [17], such as guinea pigs [18], rabbits [19], monkeys [20], and mice, with murine models being the most common [21, 22]. Although experimental murine TB cannot totally imitate human TB due to diverse factors and complexities, these models have provided valuable insights into TB pathogenesis and treatment [12, 23]. Some mouse models have been developed to characterize the cellular and molecular processes involved in bovine TB immunopathogenesis and the formation of necrotic granulomas [15, 24].

T helper 17 (Th17) cells are a subpopulation of CD4^+^ T lymphocytes that have an essential role in defense against pathogens. Th17 cells differ from Th1 and Th2 cells in their production of the interleukin-17 (IL-17) cytokine family [25], which includes six members from IL-17A to IL-17-F [26, 27] The main members, IL-17A and IL-17F, are produced by cells of the innate and adaptative immune system and activate the production of inflammatory mediators, such as tumor necrosis factor α (TNF-α), IL-1β, IL-6, granulocyte colony-stimulating factor (G-CSF), and granulocyte-macrophage colony-stimulating factor (GM-CSF). They also induce the production of chemokines, including CXCL1, CXCL5, CCL2, and CCL7, and the expression of antimicrobial peptides, which mediate the activation and recruitment of inflammatory cells such as neutrophils [28]. IL-17A is the main cytokine produced by Th17 lymphocytes [29–37] and is an important participating element of the adaptive immune system [30, 38] related to the protective immune response against mycobacterial infection [13, 36, 39–43]; however, IL-17A has been also related to the production of intense inflammation associated with necrosis [43–45] due to the recruitment, activation, and migration of neutrophils [46, 47] and the persistence of viable antigens [48–52]. Cattle experimentally infected with *M*. *bovis* strains show both CD4^+^ and CD8^+^ lymphocyte activation during the early stages of infection, as well as IL-17A gene expression that correlates with lung pathology [53].

In a previous study using a murine model of pulmonary TB in BALB/c mice, we tested selected *M*. *bovis* isolates with different genotypes obtained from diverse hosts that demonstrated different levels of virulence [54]. Strains isolated from wild animals (wild boars) were highly virulent and produced high mortality and extensive pulmonary necrosis, while strains from cattle or humans exhibited lower virulence. In the present study, we used these *M*. *bovis* isolates with different levels of virulence to determine the production of IL-17A and its contribution to protection or tissue damage (pulmonary necrosis) in BALB/c mice. In the first part of the study, the gene expression kinetics and cellular sources of IL-17A were determined. Then, in the second part, the contribution of IL-17A to immune protection and pulmonary necrosis was evaluated by suppressing IL-17A via the administration of specific blocking antibodies and determining animal survival, pulmonary bacillary loads, and the extent of tissue damage.

## Materials and methods

### Strain selection

Two strains of *M*. *bovis* with different levels of virulence were selected [54]. *M*. *bovis* strain 534 was isolated from cattle. BALB/c mice infected with this strain showed lower bacillary loads and total survival after 4 months of infection, while the highly virulent *M*. *bovis* strain 04–303 isolated from a wild boar produced high pulmonary bacillary loads and total mortality at 1 month of infection, with extensive pneumonia and massive necrosis.

These bacterial strains were grown in Middlebrook 7H9 broth (Difco Laboratories, Detroit, MI, USA) enriched with 10% albumin, dextrose, and catalase (ADC, Difco Labs) and 0.05% Tween 80, in constant agitation at 37°C and 5% CO_2_ over 21 days. Mid-log-phase cultures were stored at −70°C until their use.

### Animal handling

This study was carried out in strict accordance with the recommendations of the Mexican Constitution law “NOM-062–Z00-1999 Technical specifications for production, care and use of laboratory animals” and with the approval of the Ethical Committee for Experimentation in Animals of the National Institute of Medical Sciences and Nutrition in Mexico (CINVA, Protocol Number CINVA- PAT-1644-15/18-1).

Human endpoints were implemented in this study to prevent or alleviate animal pain or distress and substitute for more severe experimental outcomes such as advanced pathology or death [55]. All treatments were administered under sevoflurane anesthesia applied in a gas chamber, and all efforts were made to minimize suffering and pain. Animals were monitored every day, and when they showed behavioral changes, such as the inability to ambulate or maintain an upright position that prevented their easy access to food and/or water; or physical abnormalities, such as agonal breathing signs of respiratory insufficiency, severe muscular atrophy, accentuated cachexia, rough coat, or total immobilization [56], they were immediately humanely euthanized under anesthesia with intraperitoneal pentobarbital. No animals died before meeting the criteria for euthanasia.

### Experimental model of pulmonary TB

The experimental model of progressive pulmonary TB has been previously described [57]. As our previous work on these bacterial strains [54] and many other studies that evaluated the level of virulence of diverse clinical isolates were performed in male mice, the present study only used male mice [58–62]. Briefly, 6–8-week-old male BALB/c mice were anesthetized in a gas chamber using sevoflurane and infected through endotracheal instillation with 2.5 × 10^5^ colony-forming units (CFUs) in 100 μl of phosphate-buffered saline (PBS) using a blunt stainless-steel cannula with a small ball in its terminal end that was inserted through the mouth and directed to the trachea. The proper intratracheal placement of the cannula was verified by palpation of the small ball from the cannula rubbing the tracheal rings. After bacillus inoculation, the mice were maintained in a vertical position until spontaneous recovery. Infected mice per strain were maintained in groups of five in cages fitted with micro-isolators in a BSL-3 animal facility. The animals were monitored every day and humanely euthanized when they showed the behavioral changes or physical abnormalities described above. Two independent experiments were performed.

In the first part of the study, groups of 48 BALB/c mice were infected either with the *M*. *bovis* strains 534 or 04–303, and groups of six infected mice were euthanized by exsanguination under anesthesia with 56 mg/kg of intraperitoneal pentobarbital at 1, 3, 7, 14, 21, 28, 60, and 120 days post-infection. Lungs were obtained for microbiological, histopathological, and immunological studies.

### Assessment of live bacillus burdens by CFUs

Lung bacillary load was determined by counting CFUs, using the right lungs from five mice at each time point in two independent experiments. Frozen lungs were processed with a FastPrep-24 homogenizer (MP Biomedicals, Santa Ana, CA, USA) in sterile tubes with 1 ml of 0.05% PBS-Tween 80 solution. Four dilutions of each homogenate were spread onto duplicate plates containing Middlebrook 7H10 agar (Difco Labs) enriched with oleic acid, albumin, dextrose, and catalase (OADC, Difco Labs). Incubation time before colony counting was 14 and 21 days. The data points shown are the means of five animals.

### Real-time polymerase chain reaction (RT-PCR) analysis of cytokine expression

Lung lobes from three different mice per group in two different experiments were used to isolate total mRNA using the RNAeasy Mini kit (Qiagen Inc.). The quality and quantity of RNA were evaluated through spectrophotometry (260/280) and on agarose gels. Reverse transcription of mRNA was performed using 100 ng RNA, oligo-dT, and the Omniscript kit (Qiagen). RT-PCR was performed using the 7500 real-time PCR system (Applied Biosystems, Foster City, CA, USA) and Quantitect SYBR green Mastermix kit (Qiagen). Standard curves of quantified and diluted PCR products, as well as negative controls, were included in each PCR run. Specific primers for genes encoding for the housekeeping gene ribosomal protein large (Rplp0), TNF-α, IFN-γ, IL-17A, IL-23, G-CSF, IL-17R, IL-4, and IL-13 (Table 1) were designed using the program Primer Express (Applied Biosystems) [63]. Data are shown as copies of cytokine-specific mRNA relative to one million copies of mRNA encoding the housekeeping gene.

**Table 1:** Primer sequences.

Gen	Forward primer (5’-3’)	Reverse primer (5’-3’)	Product size	Application
Rplp0	CTCTCGCTTTCTGGAGGGTG	ACGCGCTTGTACCCATTGAT	108 pb	RT-PCR
TNF-α	AAATGGCCTCCCTCTCATCAGT	GATCTGAGTGTGAGGGTCTGGG	51 pb	RT-PCR
IFN-γ	CCTCAAACTTGGCAATACTCAT	GGTGACATGAAAATCCTGCAG	181 pb	RT-PCR
IL-17A	TGACCCCTAAGAAACCCCCA	GTGGAGGGCAGACAATTCTGA	123 pb	RT-PCR
IL-23	AAAGGATCCGCCAAGGTCTG	GGAGGTGTGAAGTTGCTCCA	110 pb	RT-PCR
G-CSF	GAGGCGCATGAAGCTAATGG	TCCAGGGACTTAAGCAGGAAG	144 pb	RT-PCR
IL-17R	AGCAGCTGCCTAAATGACTGT	CTGCAACTGGCTTGGGAACT	88 pb	RT-PCR
IL-4	GCAGCTTATCGATGAATCCAGG	CGTCCTCACAGCAACGGAGA	181 pb	RT-PCR
IL-13	CAAGGCCCCCACTACGGT	CGTGGGGAAACAGTTGCTTT	101 pb	RT-PCR

**Table 1: Primer sequences.** List of specific primers designed using the program Primer Express (Applied Biosystems), specifying product size and application.

### Morphometry, immunohistochemistry (IHC), and digital pathology

Four left lungs from each experimental group and time point were perfused with absolute ethanol, fixed for 24 hr, and embedded in paraffin. Sections 4 μm thick were mounted on glass slides, deparaffinized, and stained with hematoxylin and eosin for histological analysis. For the quantification of tissue damage or granuloma area by automated morphometry, three section levels 10 μ apart per mouse were used. The total lung surface and the area affected by pneumonia and necrosis were measured using a Q-win Leica 500 Image Analysis System (Leica Q-win, Cambridge, UK) and reported as the percentage of the affected lung surface. Histologically, pneumonia was identified by alveolar spaces and alveolar-capillary interstitium occupied by inflammatory cells, while necrosis corresponded to lung areas substituted by amorphous eosinophilic cytoplasmic debris mixed with spherical condensed or fragmented basophilic material that corresponded to condensed or nuclear fragments, respectively [14]. The area in square microns of all granulomas identified in the three sections, usually six to 10, was measured using the same automated morphometry system.

The same paraffin-embedded tissue sections were used for IL-17A detection to determine local cytokine production by IHC. Briefly, slides were incubated with rabbit IgG anti-mouse IL-17A (Santa Cruz Biotechnology, Santa Cruz, CA, USA; sc-7927) diluted 1:100 in the ImmunoDetector Protein Blocker/Antibody Diluent (Bio SB). The reaction was followed by hematoxylin counterstaining. To determine cytokine production by digital pathology, different immunostained areas (pneumonia, granuloma, and perivascular, interstitial, and peribronchial inflammation) were measured in three left lungs from each experimental group using digital automated morphometry as previously described [59, 64–66]. The IHC-stained sections were digitized at 40x magnification using an Aperio Scanscope CS (Aperio, Vista CA, USA), obtaining 40 images with a spatial resolution of 0.45 μm/pixel. The images were reviewed using an ImageScope (Aperio). Once the areas were recorded (500 mm for each tissue), they were sent for automated image analysis using the Spectrum software V11.1.2.752 or the Pixel Count v9 algorithm (Aperio). For the within-tissue stain intensity, an algorithm was developed to quantify the cytoplasmic protein expression. The output from the algorithm comprised several quantitative measurements (intensity, concentration, and percentage of positive staining). The staining intensity was categorized as 0 (no staining), 2+ (moderate), and 3+ (strong). The average of different stained areas was analyzed and represented as total intensity expression (pixels/μm^2^), which corresponds to the estimated concentration of the selected cytokine.

### Flow cytometry

In three different mice per group, cells were isolated from excised lungs and minced with scissors in 1 mL of digestion buffer (RPMI-1640, 1 mg/mL collagenase IV, Sigma-Aldrich, Saint Louis, MO, USA), for 1 hr. Tissue fragments were disaggregated by passing through needles of different gauges (18G and 21G) and then centrifuged (1,200 rpm, 5 min, 4°C). Red blood cells were eliminated using lysis buffer, and the remaining cells were filtered through a 50 μm pore size nylon filter (BD Biosciences). Dead cells were excluded by trypan blue staining and live cells were resuspended in PBS containing 2% bovine serum albumin (Sigma-Aldrich). A total of 5 × 10^6^ cells/mL were stimulated at 37°C, 5% CO_2_, in a humidified atmosphere for 6 hr with Cell Stimulation Cocktail (plus protein transport inhibitors, eBioscience-Thermo Fisher Scientific). Then, the cells were incubated for 10 min at 4°C with 0.5 μg of anti-mouse CD16/CD32 (2.4G2) antibody (Ab; FcR blocker, Tonbo, San Diego CA, USA; 70–0161) followed by the addition of 0.5 μg per 10^6^ cells of the viability Ghost Dye-UV 450 (Tonbo; 13–0868) and the extracellular Abs CD3-redFluor 710 (17A2, Tonbo; 800032), CD4-BV510 (Biolegend, San Diego, CA, USA; 100559), CD8-BV711 (Biolegend; 100759), CD11b-FITC (eBioscience; 11-0112-82), or Ly-6G-V450 (1A8, Tonbo; 75–1276), all diluted 1:100 in PBS. For intracellular labeling, cells were permeabilized and fixed using the Foxp3/Transcription Factor Staining Buffer Set (eBiosciences; 00-5523-00) for 30 min at 4°C and then stained with the intracellular Abs IFN-γ-BV605 (Biolegend; 505839), IL-4-PE (BD Biosciences; 554389), or IL-17A-PECF594 (BD Biosciences; 562542) for 30 min at 4°C in the dark. Samples were analyzed in a BD-LSRFORTESSA^™^ flow cytometer. Data were analyzed by FlowJo^™^ software (Tree Star, Inc., San Carlos, CA, USA). First, the lymphocyte zone was selected from the forward scatter height (FSC-H) vs forward scatter height (FSC-A) gate, and 100,000 live cells were selected from CD3 vs CD4 to obtain the percentage of CD4^+^ IL17A^+^, CD4^+^ IL4^+^, or CD4^+^ IFN-γ^+^ lymphocytes. Then, the granulocyte zone was selected from the FSC-H vs FSC-A gate, and 100,000 live CD11b^+^ cells were selected to obtain the percentage of Ly6G^+^ IL-17A^+^ neutrophils.

### IL-17A neutralization

To evaluate the immunological contribution of IL-17A to *M*. *bovis* infection, particularly in the development of necrosis and protection against infection, groups of BALB/c mice were infected with strain 04–303 and treated with 25 μg of IL-17A neutralizing antibodies (R&D Systems, Minneapolis, MN, USA; MAB421) or isotype control antibodies (R&D Systems; MAB006), administered directly to the lungs by endotracheal instillation every other day between 13 and 19 days post-infection. The animals were euthanized on Days 21 and 24. Mice infected with the *M*. *bovis* 534 attenuated strain were equally treated from 6 to 12 days post-infection and euthanized on Days 7 and 14 after infection. The treatment intervals were chosen in relation to the highest IL-17A production in the lungs.

The lungs of four mice per time point were immediately removed and fixed by perfusion with ethylic alcohol and embedded in paraffin, and sections stained with hematoxylin/eosin were used for the quantification of tissue damage (necrosis, pneumonia) or granuloma area by automated morphometry, as described above.

Right lungs from five mice at each time point in two independent experiments were used for CFU determination by the method described above.

Lung lobes from three different mice per group were used to isolate mRNA and determine the expression of cytokines by RT-PCR following the procedure described above. Table 1 shows the specific primers for the cytokines determined that were designed using the program Primer Express (Applied Biosystems) [63]. The quantities of the specific mRNA in the sample were measured according to the corresponding gene-specific standard. The mRNA copy number of each cytokine was related to one million copies of mRNA encoding the housekeeping gene.

### Statistical analysis

All data were analyzed using ANOVA followed by the Bonferroni multiple comparison test (GraphPad Prism, GraphPad software version 8.0.1, USA). For comparing experimental groups, 95% confidence interval values were considered significant when p<0.05.

## Results

### *M*. *bovis* infection increases IL-17A production

**Fig 1:**
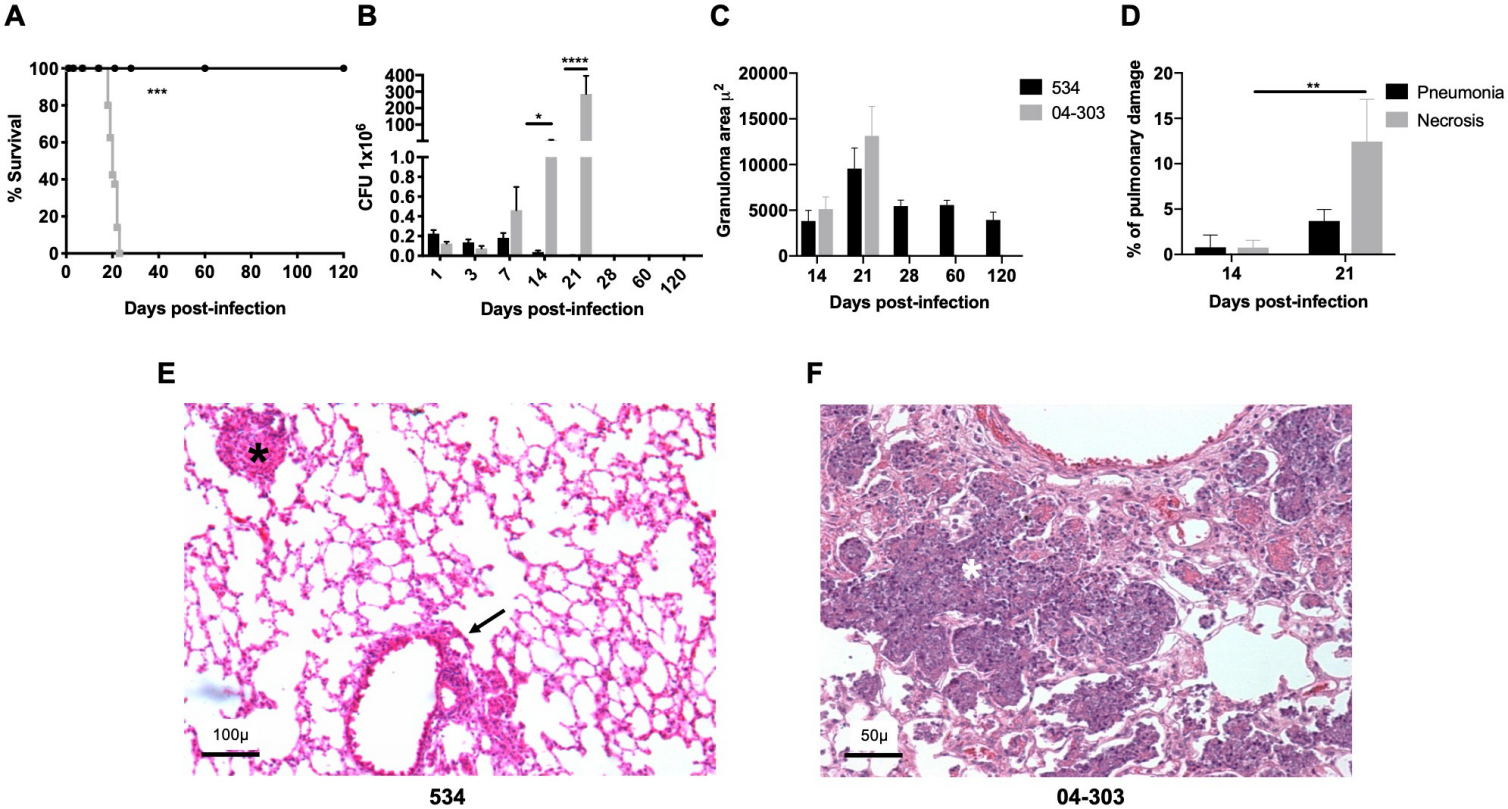
Comparative virulence of *M*. *bovis* strains 534 and 04–303. Groups of 48 BALB/c mice were infected either with the *M*. *bovis* strains 534 or 04–303, and groups of six infected mice were euthanized at 1, 3, 7, 14, 21, 28, 60, and 120 days post-infection, in two different experiments. A) Survival rates of BALB/c mice infected with *M*. *bovis* isolates. Strain 534 permitted total survival, while strain 04–303 produced total mortality at 24 days of infection. B) Lung bacillary loads of mice infected with *M*. *bovis* strains 534 or 04–303 (n = 5 per timepoint /group). C) Granuloma size of lungs infected with *M*. *bovis* strains 534 or 04–303 (n = 4 per timepoint /group). D) Percentage of lung surface area affected by necrosis or pneumonia of mice infected with the virulent *M*. *bovis* strain 04–303 (n = 4 per timepoint /group). One representative example is shown from two independent experiments. Data were analyzed by ANOVA followed by the Bonferroni multiple comparison test. Asterisks represent statistical significance (*p<0.05, **p<0.005, ***p<0.001, ****p<0.0001) between strains. Representative lung micrographs (hematoxylin/eosin staining) on Day 21 post-infection. E) Strain 534 induced slight inflammation around the airways (arrow), blood vessels, and interstitium, as well as granulomas (asterisk; 200x magnification). F) Infection with strain 04–303 produced extensive pneumonia with massive necrosis (white asterisk; 100x magnification).

Groups of BALB/c mice were infected intratracheally with 2.5 × 10^5^ CFUs of the 534 or 04–303 *M*. *bovis* strains. All the animals infected with strain 534 survived after 4 months of infection with very low bacterial loads, granuloma formation from Day 14, and lower inflammatory infiltrate levels around airways, blood vessels, and the alveolar-capillary interstitium without pneumonia (Fig 1). In contrast, mice inoculated with strain 04–303 started to die after 16 days of infection, and all animals died by Day 23, with a progressive increase in bacillus loads rising to a peak on Day 21 and areas of extensive pneumonia and necrosis at the same time point (Fig 1). These results confirmed the previously reported attenuation of strain 534 and the high virulence of strain 04–303 [54].

**Fig 2:**
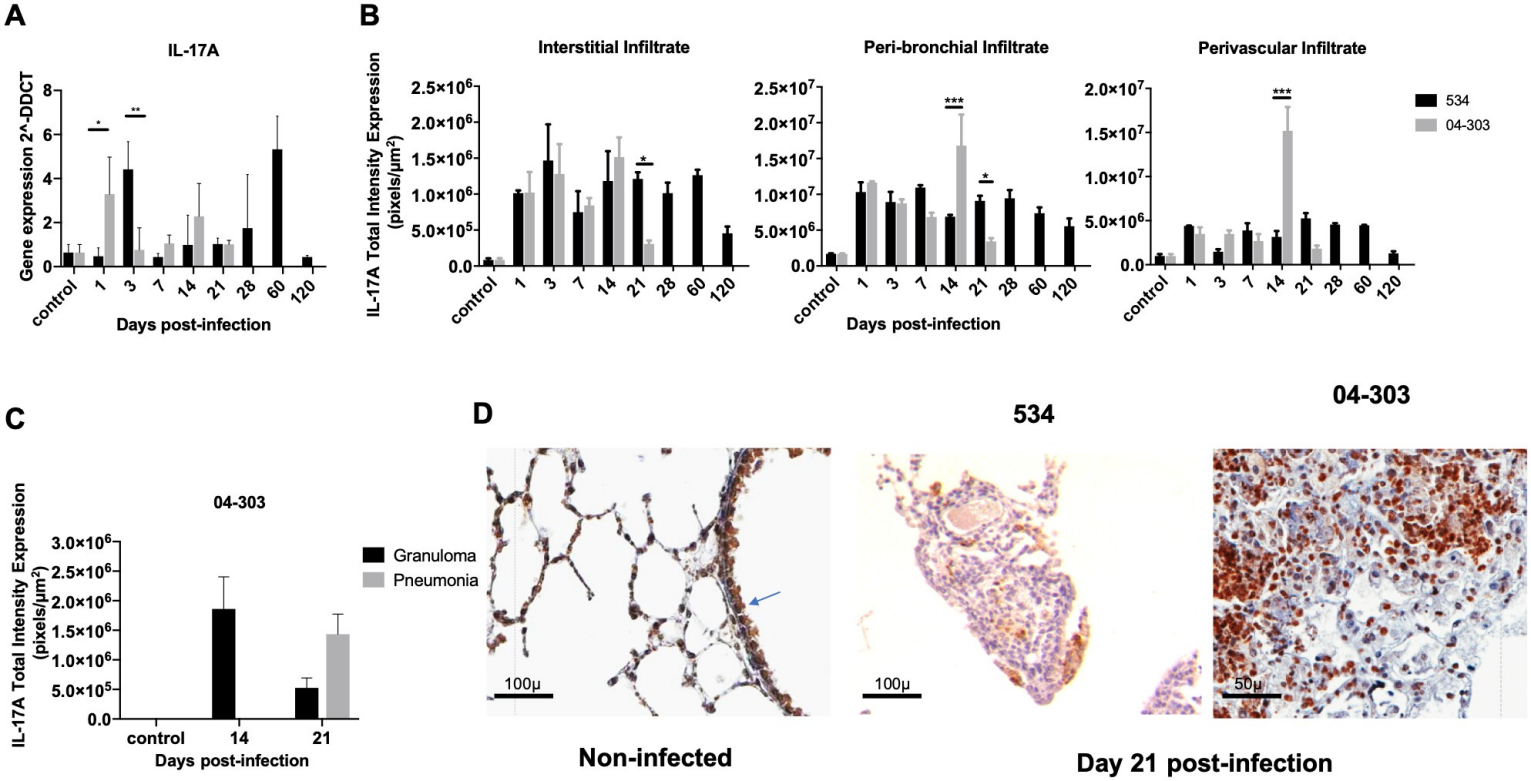
Kinetics of IL-17A during infection with *M*. *bovis* strains 534 and 04–303. A) Quantitative expression of IL-17A gene transcription determined by RT-PCR in the infected lungs of mice with *M*. *bovis* attenuated strain 534 or highly virulent strain 04–303 (n = 3 per timepoint /group). B) IL-17A total intensity expression in lungs assessed by digital pathology during infection with the indicated *M*. *bovis* strains (n = 3 per timepoint /group). The immunohistochemically stained sections were digitized and analyzed using an Aperio Scanscope CS. An algorithm was developed to quantify the cytoplasmic protein expression related to stain intensity, and the average of different stained areas was analyzed and represented as total intensity expression (pixels/μm^2^), which corresponds to the estimated concentration of the selected cytokine in the indicated lung compartment. C) Total intensity expression of IL-17A in granulomas and pneumonia induced by strain 04–303 (n = 3 per timepoint /group). One representative example is shown from two independent experiments. Data were analyzed by ANOVA followed by the Bonferroni multiple comparison test. Each graph represents the means ± SE of IL-17A cellular detection of three mice for each day of the time course. Asterisks represent statistical significance between strains (*p<0.05, **p<0.01, **p<0.001). D) Representative micrographs of IL-17A immunostaining in non-infected lungs showing positive labeling in the bronchial epithelium (arrow) and some lymphocytes, and, on Day 21 post-infection, showing numerous positive cells in granuloma induced by infection with strain 534 and in pneumonic areas induced by strain 04–303 near necrotic cells (200x and 400x magnification, respectively).

To evaluate the kinetics of IL-17A production during *M*. *bovis* lung infection, cytokine gene expression was determined by RT-PCR and protein production and cellular sources through IHC/digital pathology and flow cytometry (Fig 2). Our results showed low gene expression of IL-17A in normal non-infected lungs, with infection with either strain increasing its expression. The lungs infected with attenuated *M*. *bovis* strain 534 showed high IL-17A mRNA expression on Day 3, followed by a decrease in expression during the early stage, and a second increase on Day 60, when very low CFUs were detected (Fig 2A). In contrast, infection with the highly virulent strain 04–303 induced two peaks of IL-17A mRNA expression, on Days 1 and 14 of infection (the latter also being the day on which pneumonia started), and 1 week later, expression was extensive and associated with necrosis and high mortality (Figs 1 and 2).

Using IHC, we determined the cellular sources of IL-17A, and by digital pathology, we quantified IL-17A protein production in the different compartments affected by inflammation (alveolar-capillary interstitium, perivascular and peribronchial areas). Non-infected lungs showed occasional IL-17A-positive cells with lymphocyte morphology around blood vessels and airways, with strong positivity in the epithelium of some bronchial ducts (Fig 2B), while lungs infected with the low-virulence strain 534 showed an increase of IL-17A total intensity expression in the interstitial, peribronchial and perivascular inflammatory infiltrate, with lymphocytes being the most frequent positively stained cells from Days 1 to 60, returning to basal levels on Day 120; numerous positive lymphocytes were detected in granulomas, and bronchial epithelium maintained constant positivity throughout the infection (Fig 2C).

Lungs infected with the highly virulent strain 04–303 showed an increase of IL-17A immunostained cells in the interstitial, peri-bronchial, and perivascular inflammatory infiltrate during the first and second weeks post-infection, with a peak after 14 days of infection (Fig 2B). Immunostained cells showed lymphocyte and macrophage morphology. IL-17A total intensity expression was reduced on Day 21 post-infection in these pulmonary compartments and in granulomas and pneumonia, where some cells with macrophage and lymphocyte morphology were positive (Fig 2C and 2D).

Interestingly, although the IL-17-A RNA levels for both strains were similar on Day 1, higher protein production was detected in lungs infected with the low virulence strain; perhaps part of the mRNA and protein was not detected due to the extensive tissue necrosis.

**Fig 3:**
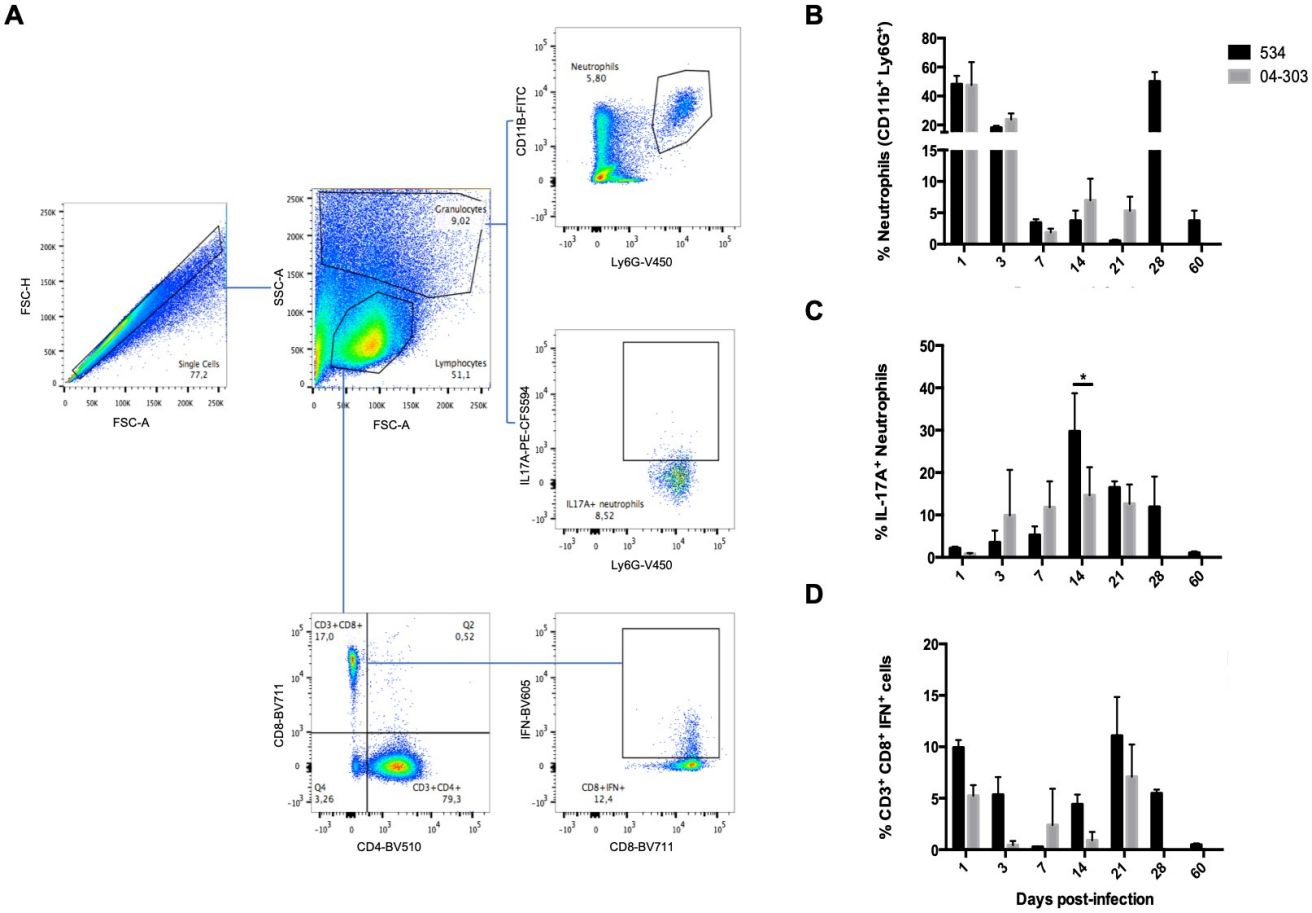
Kinetics of neutrophils and CD8^+^ cells in the lungs of mice infected with attenuated or highly virulent *M*. *bovis* strains. A) Gating strategy of representative flow cytometry samples of lung lobes from three different BALB/c mice per group infected with *M*. *bovis* strains 534 or 04–303 (n = 3 per timepoint /group). First, the granulocyte zone was selected from the FSC-H vs FSC-A gate, and 100,000 live CD11b^+^ cells were selected to obtain the percentage of Ly6G^+^ IL-17A^+^ neutrophils. Then, the lymphocyte zone was selected from the FSC-H vs FSC-A gate, and 100,000 CD3^+^ live cells were selected to obtain the percentage of CD8^+^ IFN-γ^+^ lymphocytes. B) Frequency of neutrophils (CD11c^+^ Ly6G^+^). C) Frequency of IL-17A^+^ neutrophils. D) Frequency of CD8^+^ T lymphocytes (CD3^+^ CD8^+^ IFN^+^). One representative example is shown from two independent experiments. Data were analyzed with ANOVA followed by the Bonferroni multiple comparison test. Values are expressed as mean ± SE. Asterisks represent statistical significance between strains (*p<0.05).

To determine the profile of inflammatory cells and IL-17A cellular sources in the lungs after infection with either poorly or highly virulent strains of *M*. *bovis*, lungs were collected at different days post-infection to obtain cellular suspensions and determine the number of CD11B^+^ Ly6G^+^ neutrophils, IL-17A^+^ neutrophils, and CD3^+^ CD8^+^ IFN^+^ lymphocytes (cytotoxic lymphocytes) by cytofluorometry (Fig 3). Initially, we selected the singlets in FSC-H against FSC-A; then, we delineated a gate to include neutrophils and then a sub-gate to exclude dead cells, which allowed us to determine the percentage of each cell subtype (Fig 3A).

CD11B^+^ Ly6G^+^ neutrophils were the predominant cells in the lungs of mice infected with either strain during early infection (Days 1 and 3) (Fig 3B). This percentage was reduced in lungs infected with the low-virulence strain 534 and increased again on Day 28 post-infection, which was the last experimental time point with cultivable CFUs (Fig 1B). The percentage of IL-17A^+^ neutrophils was high during days of maximal bacterial growth control in the low-virulence strain infection. In contrast, the lungs infected with the highly virulent *M*. *bovis* strain 04–303 showed an increase in the percentage of CD11B^+^ Ly6G^+^ neutrophils on Days 14 and 21, with the latter being associated with extensive pulmonary necrosis, and the number of IL-17A^+^ neutrophils was maintained during the whole course of infection, with a slight increase in the days related with pulmonary necrosis (Fig 3C). The percentage of CD8^+^ IFN^+^ on Days 12 and 21 was higher in the low virulence strain infection in comparison with the highly virulent *M*. *bovis* strain 04–303 (Fig 3D).

**Fig 4:**
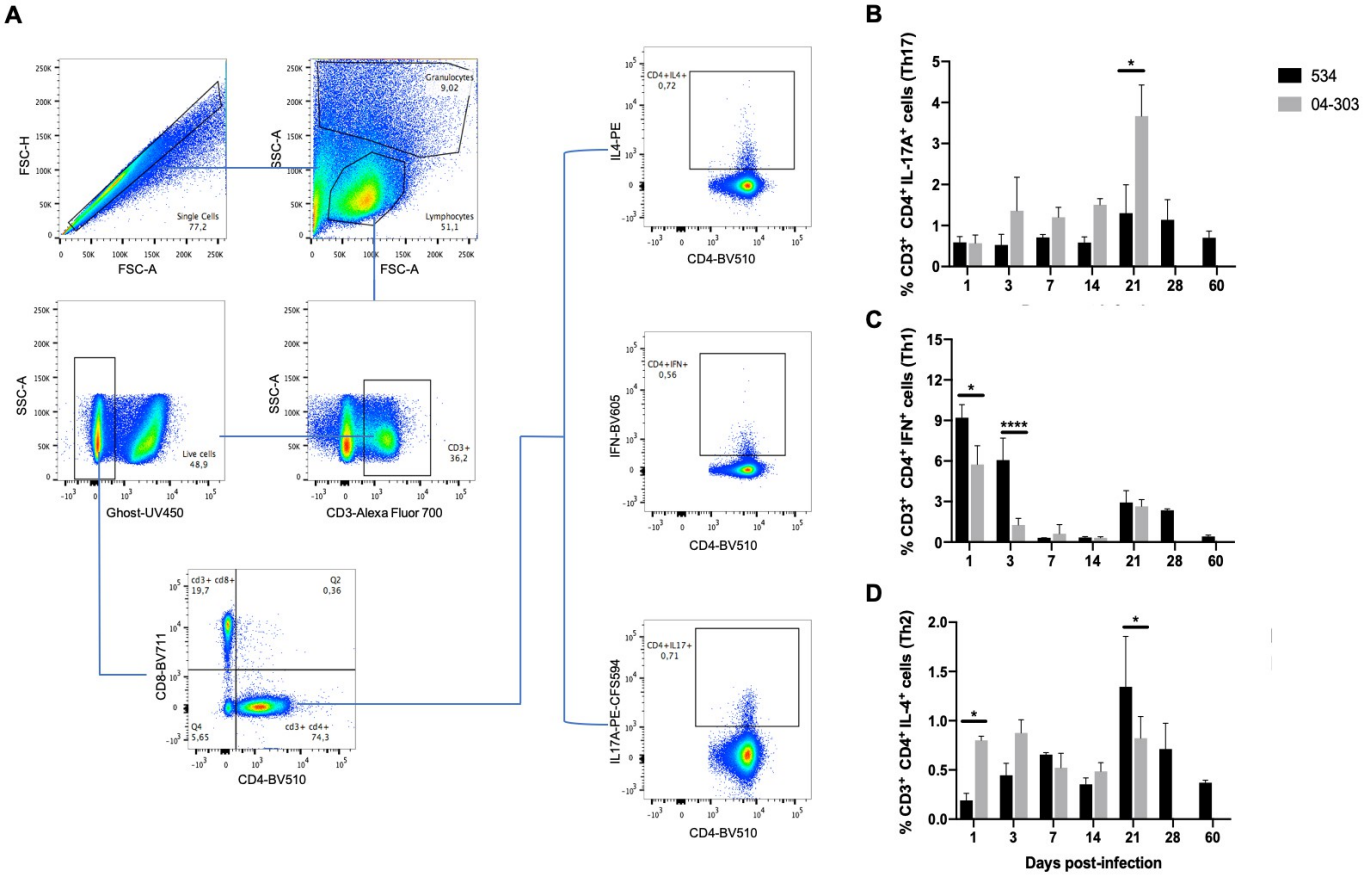
Kinetics of Th17, Th1, and Th2 cells in the lungs of mice infected with attenuated or highly virulent *M*. *bovis* strains. A) Gating strategy of representative flow cytometry samples of lung lobes from three different BALB/c mice per group infected with *M*. *bovis* strains 534 or 04–303 (n = 3 per timepoint /group). The lymphocyte zone was selected from the FSC-H vs FSC-A gate, and 100,000 alive cells were selected from the CD3 vs CD4 gate to obtain the percentage of CD4^+^ IL17A^+^, CD4^+^ IL4^+^, or CD4^+^ IFN-γ^+^ lymphocytes. B) Percentage of Th17 cells (CD3^+^ CD4^+^ IL-17A^+^). C) Percentage of Th1 lymphocytes (CD3^+^ CD4^+^ IFN^+^). D) Percentage of Th2 lymphocytes (CD3^+^ CD4^+^ IL-4^+^). One representative example is shown from two independent experiments. Data were analyzed with ANOVA followed by the Bonferroni multiple comparison test. Values are expressed as mean ± SE. Asterisks represent statistical significance between strains (*p<0.05, ****p<0.0001).

Similarly, the profile of CD3^+^ CD4^+^ IFN^+^ (Th1), CD3^+^ CD4^+^ IL-4^+^ (Th2), and CD3^+^ CD4^+^ IL-17A^+^ (Th17) lymphocytes in the lungs of infected mice was determined (Fig 4). Initially, we selected the singlets in FSC-H against FSC-A; then, we delineated a gate to include lymphocytes and then a sub-gate to exclude dead cells, which allowed us to determine the percentage of each cell subtype (Fig 4A). Animals infected with the low-virulence strain 534 showed a low and stable percentage of CD3+, CD4+, and IL-17A+ lymphocytes during the first and second weeks of infection. This percentage increased two-fold on Days 21 and 28 of infection along with efficient bacterial growth control (Fig 4B). Mice infected with the high-virulence strain 04–303 showed a rapid and stable increase of CD3^+^, CD4^+^, and IL-17A^+^ cells from Days 3 to 14 after infection. On Day 21, the percentage increased three-fold along with extensive necrosis (Fig 4B). Th1 cells increased during the initial days post-infection, as well as on Days 21 and 28 (Fig 4C), together with a subtle increase in the percentage of Th2 cells when bacterial growth in the lungs of mice infected with the low-virulence strain decreased (Fig 4D). In comparison, in the lungs infected with the highly virulent *M*. *bovis* strain 04–303, Th1 and Th2 cells showed constant percentages during all the studied time points (Fig 4C and 4D). Thus, IL-17A producer cells were more numerous in mice infected with the attenuated strain when efficient control of bacilli growth was seen and at the time of maximal necrosis in animals infected with the highly virulent strain. This immune response was considered a good opportunity to evaluate the contribution of IL-17A to either protection against or tissue damage induced by *M*. *bovis*, and this was explored with the administration of IL-17A blocking antibodies.

### The effect of blocking IL-17A activity in mice infected with attenuated or highly virulent strains of *M*. *bovis*

**Fig 5:**
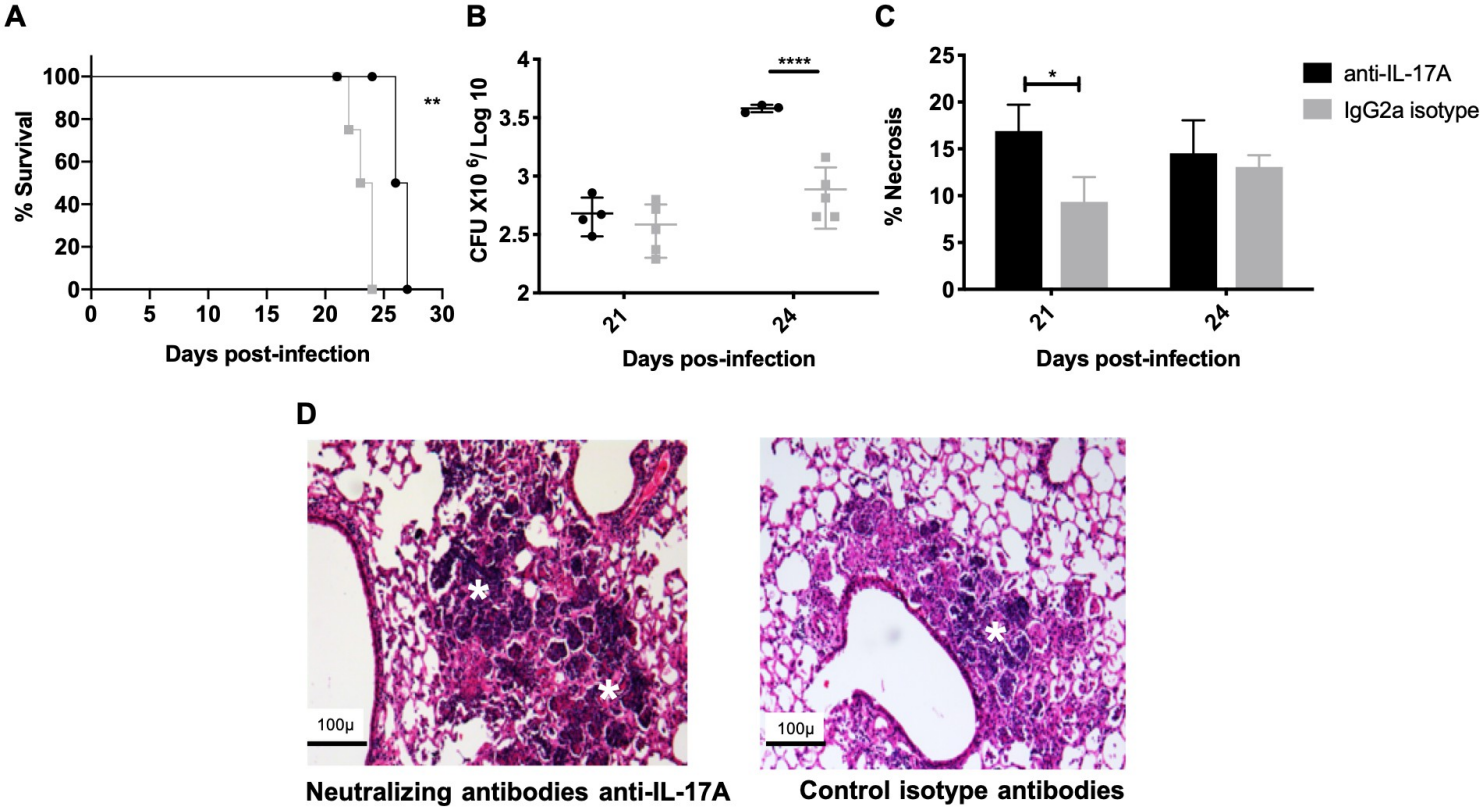
Effect of IL-17A blocking antibodies on mice infected with the highly virulent strain 04–303. BALB/c mice were infected with *M*. *bovis* strain 04–303 and treated with 25 μg of IL-17A neutralizing antibodies (R&D Systems; MAB421) or isotype control antibodies (R&D Systems; MAB006) administered directly to the lung by endotracheal instillation every third day from Days 13 to 19 post-infection. A) Survival curves: mice treated with blocking IL-17A antibodies show significantly higher mortality. B) Significantly higher bacillary loads are seen in mice after eight days of start anti-IL-17A antibody administration (n = 5 per timepoint /group). C) Automated morphometry measurement of necrosis confirms more tissue damage in treated than in control mice (n = 4 per timepoint /group). D) Representative micrographs of the lungs of treated and non-treated mice with neutralizing IL-17A antibodies: the mouse lungs after five days of treatment show more extensive tissue damage (white asterisks indicate necrotic areas). Bacillary loads and morphometry curves were measured in five animals per time point in two different experiments. One representative example is shown from two independent experiments. Data were analyzed by applying ANOVA followed by the Bonferroni multiple comparison test. Asterisks represent statistical significance between treatments; values are expressed as mean ± SE (*p<0.05, **p<0.005, ****p<0.0001).

To determine if IL-17A participates in the pulmonary damage induced by the highly virulent *M*. *bovis* strain 04–303, infected mice were treated with 25 μg of IL-17A neutralizing antibodies (R&D Systems, Minneapolis, MN, USA; MAB421) or isotype control antibodies (R&D Systems; MAB006), directly administered to the lung by endotracheal instillation every other day from Days 13 to 19 post-infection, just before the development of pulmonary necrosis started (Fig 5). The survival curve was constructed in a group of six untouched animals (Fig 5A). Animals were euthanized on Days 21 and 24 post-infection, and lungs were used to evaluate the bacillus burdens and histopathology comparing control and treated mice (Fig 5B–5D). In these mice, neutralization of IL-17A produced a significant decrease in survival, an increase in necrosis on Day 21 and significantly higher bacilli burdens on Day 24 post-infection, in comparison with the control group that received isotype antibodies (Fig 5).

**Fig 6:**
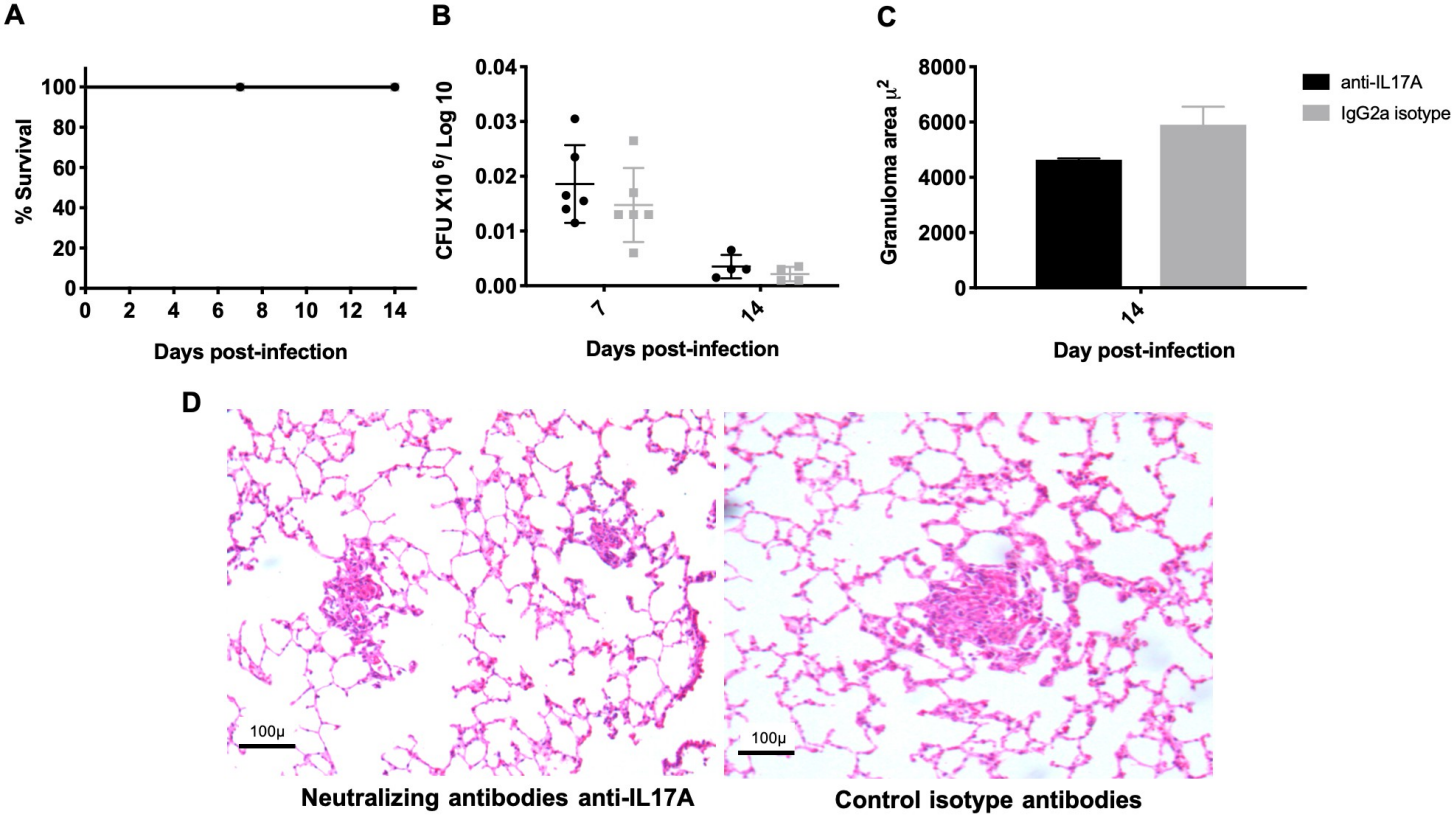
Effect of neutralizing IL-17A in the lungs of infected mice with a low-virulence *M*. *bovis* strain. BALB/c mice were infected with low-virulence *M*. *bovis* strain 534 and treated with 25 μg of IL-17A neutralizing antibodies (R&D Systems; MAB421) or isotype control antibodies (R&D Systems; MAB006) administered directly to the lung by endotracheal instillation every third day from Days 6 to 12 post-infection. A) Survival rates of infected and treated mice. B) Lung bacillary loads of infected and treated mice (n = 5 per timepoint /group). C) Granuloma size of infected and treated lungs (n = 4 per timepoint /group). D) Representative micrographs of infected and treated mice with anti-IL-17A or IgG2a isotype on Day 7 post-infection, showing smaller granulomas and lower inflammatory infiltrate around the airways and alveolar-capillary interstitium in mice treated with IL-17A blocking antibodies (micrographs of hematoxylin/eosin staining, 200x magnification). One representative example is shown from two independent experiments Data were analyzed by applying ANOVA followed by the Bonferroni multiple comparison test.

In comparison with control mice that received isotype antibodies, IL-17A neutralization in mice infected with the low-virulence *M*. *bovis* strain 534 from Days 6 to 12 post-infection, when the highest IL-17A production in the lung was seen, did not affect mouse survival but produced a significant increase in bacillary burdens after seven days of infection and a non-significant decrease in inflammatory infiltrated and granuloma areas (Fig 6). These results suggest that IL-17A in the infection with either low or high virulent *M*. *bovis* strains contributes to cellular recruitment and bacillary growth control, which is apparently more associated with immune protection than the induction of necrosis as previously reported [42].

### Cytokine profile after the neutralization of IL-17A in mice infected with highly virulent *M*. *bovis*

**Fig 7:**
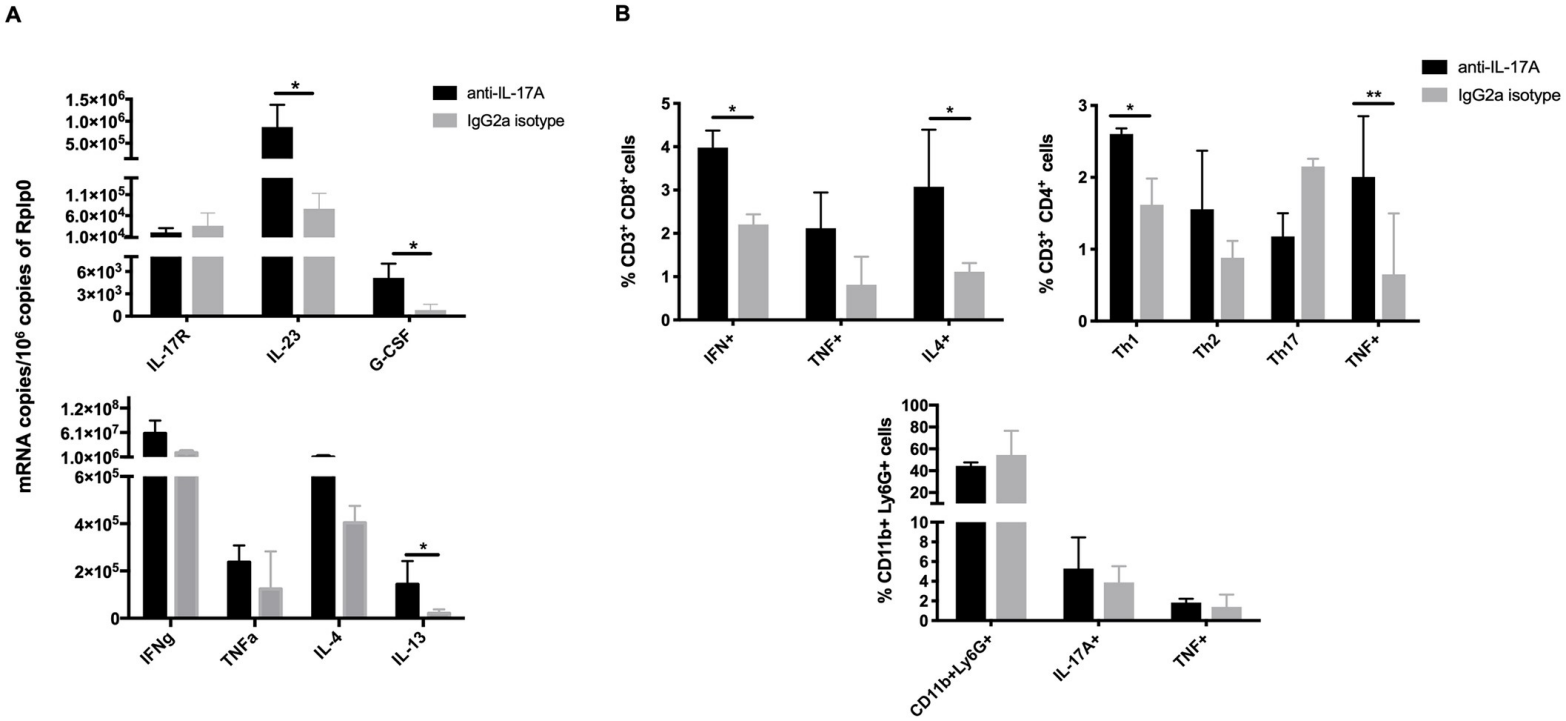
Cytokine production and T cell subsets after infection with highly virulent *M*. *bovis* strain 04–303 and IL-17A neutralization. A) Quantitative gene expression of the indicated cytokines was determined by RT-PCR using total RNA isolated from the lungs of infected mice treated with IL-17A blocking antibodies and control animals that received IgG isotype. Blocking IL-17A induced significantly higher expression of IL-23, G-CSF, and the Th2 cytokine IL-13 than in control mice and non-significantly higher transcription of the pro-inflammatory cytokines IFN-γ and TNF-α and the Th2 cytokine IL-4 (n = 3 per timepoint /group). B) Frequency of Th1 lymphocytes (CD3^+^ CD4^+^ IFN^+^), Th2 lymphocytes (CD3^+^ CD4^+^ IL-4^+^), Th17 lymphocytes (CD3^+^ CD4^+^ IL-17A^+^), CD3^+^ CD4^+^ TNF^+^ lymphocytes, CD8^+^ T lymphocytes (CD3^+^ CD8^+^), IFN^+^, TNF^+^, and IL-4^+^, and neutrophils (CD11c+ Ly6G+), IL-17A+, and TNF+ (n = 3 per timepoint /group). One representative example is shown from two independent experiments. Data were analyzed with ANOVA followed by the Bonferroni multiple comparison test. Each graph represents the means ± SE of at least three mice for each of the time points. Asterisks represent statistical significance between groups (*p<0.05, **p<0.01).

Considering the increase of necrosis and bacillary load in the lungs of mice infected with the highly virulent strain 04–303 treated with the IL-17A neutralizing antibodies, we evaluated through RT-PCR the expression of other cytokines related to protection or necrosis in *M*. *bovis* infection on Day 21 of infection (Fig 7). In comparison with the control isotype-treated group, the gene expression of IL-23 and G-CSF was significantly higher in the mice treated with IL-17A neutralizing antibodies (Fig 7A). Other cytokines that have been associated with necrosis, such as IL-4, IL-13, and TNF-α [67], were more highly expressed in the treated group than in control mice (Fig 7A).

The percentage of neutrophils and Th1, Th2, Th17, and CD8^+^ lymphocytes positive for IL-17A in lungs infected with *M*. *bovis* strain 04–303 and treated with neutralizing IL-17A antibodies or isotype antibodies was determined by cytofluorometry (Fig 7B). Animals treated with IL-17A blocking antibodies showed a significant increase in the percentage of Th1/TNF-α-positive CD4 lymphocytes and IFN-γ- and IL-4-positive CD8^+^ lymphocytes. No differences were observed in the percentage of neutrophils in either of the two treated groups (Fig 7B).

The elevation of the gene expression of these cytokines and T cells could be a compensation response, considering that IL-17A and its effects are blocked, which could induce an increase in the mRNA production of the selected protective cytokines and T cell subsets.

## Discussion

IL-17A is a cytokine that plays a crucial role in the immune protective response against bacterial and fungal infections [68–71]. Indeed, the crucial role of IL-17A in the innate immune response against extracellular organisms has been confirmed based on its capacity to activate neutrophil recruitment [72]. On the other hand, several studies have shown that IL-17A can also contribute to the development of tissue damage produced by diverse pathological conditions, such as autoimmune diseases [73–75], liver and kidney diseases [76, 77], and chronic bacterial infection [78]. In the present work, two *M*. *bovis* isolates with different levels of virulence and high production of IL-17A in infected BALB/c mice were used to study this important cytokine’s contrasting functions.

Strain 04–303 is highly virulent and produces sudden pneumonia with extensive necrosis, along with high pulmonary bacillary load and mortality, while *M*. *bovis* strain 534 is extremely attenuated [54]. We found IL-17A-positive epithelial pulmonary cells and inflammatory cells in mice infected with either poorly or highly virulent *M*. *bovis* strains from the first day of infection, which correlate with the innate function of IL-17A in the recruitment of immune cells and the induction of antimicrobial peptides during the adaptive immune response against mycobacteria [79, 80]. Th17 lymphocytes have been reported as the most important source of IL-17A, related to the induction of chemokines CXCL5 and MIP-2 as important factors in neutrophil recruitment [81]. Indeed, IL-17A, in synchrony with TNF-α, is reported to induce the secretion of chemokines and cytokines such as IL-6 and IL-23 in endothelial cells associated with neutrophil recruitment [82, 83], which also has been related to the promotion of granuloma formation, not only in *M*. *tuberculosis* infection but also in experimental paracoccidioidomycosis [84, 85]. Our results suggest that IL-17A has a protective role during *M*. *bovis* pulmonary infection, considering that mice infected with the low-virulence strain 534 highly expressed IL-17A, and when infected mice were treated with neutralizing antibodies against this cytokine, they showed smaller granulomas and less inflammatory infiltrate. The protective activity of IL-17A in mice infected with the highly virulent strain 04–303 was more evident, considering that animals infected with this strain also showed high production of IL-17A and exhibited a significant increase in bacillary load and mortality when IL-17A was blocked by the direct administration of neutralizing antibodies to the lungs. Thus, these observations confirmed that IL-17A is important for granuloma formation, considering that one of its main functions is cell recruitment, which contributes to the formation of these structures that participate in the control of bacillary proliferation and spread of infection, agreeing with previously reported observations in murine models of TB using *M*. *bovis* vaccine strains [42, 86], low-virulence *M*. *tuberculosis* strains [87], or even the highly virulent Beijing *M*. *tuberculosis* strain [88].

As mentioned before, IL-17A has been related to tissue damage in some chronic granulomatous pulmonary infections; for example, the excessive proinflammatory activity of IL-17A-mediated cytokine production was found to be harmful in a model of *Paracoccidioides brasiliensis*-infected mice treated with a heat shock protein [89]. Additionally, the repeated administration of BCG after *M*. *tuberculosis* infection induced numerous IL-17A-producing cells, with a significant increase in inflammation and necrosis in the lungs of infected mice [49]. In this regard, IL-17A-induced neutrophil recruitment has been demonstrated to contribute to tissue damage and increase of the infectious agent in chronic pulmonary infections. Indeed, some reports associate the main function of IL-17A with the massive recruitment of neutrophils [45–47, 90, 91], which is related to excessive inflammation and the consequent development of tissue damage [40, 43, 52]. Although IL-17A is a cytokine related to triggering pulmonary pathology in the presence of the antigen persistence of mycobacterial compounds, we observed that animals infected with a highly virulent strain of *M*. *bovis* that showed large patches of tissue necrosis in the lungs, along with high levels of IL-17A mRNA and protein in the days before the development of tissue damage, showed even more extensive necrosis when IL-17A was blocked by specific neutralizing antibodies.

Some studies have correlated the lung pathology in cattle experimentally infected with field *M*. *bovis* strains with IL-17A gene expression [53]. In contrast, the IL-17A response was also highly described as a cytokine biomarker of infection and protection in the immune response to bovine TB [92].

Our findings show that pulmonary necrosis is not totally mediated by the recruitment of neutrophils through IL-17A. There must be alternative mechanisms that trigger this type of lung tissue damage. Therefore, the question of which molecules and mechanisms are key in the development of necrosis in lung tissue during pulmonary TB remains open. Earlier, our BALB/c model showed that IL-4 has some detrimental effects on the antibacterial efficacy of the Th1 response and affects the toxicity of TNF-α and fibrosis [93], suggesting that TNF-α may have a paradoxical role; in the presence of Th1 cytokines, it may act as a macrophage-activating factor, but in mixed Th1/Th2 responses, it may cause tissue damage [94]. In addition, some studies have related the IL-13/IL-4R axis of Th2 cells to the development of tissue damage mediated by increased arginase and the consequent activation of alternative macrophages, which are incapable of controlling the bacterial load and the destruction of lung tissue [67]. Interestingly, these Th2 cytokines were overexpressed in the lungs of infected mice infected with the highly virulent strain and treated with IL-17A neutralizing antibodies.

Th17 lymphocytes predominantly produce IL-17A, a member of the IL-17 family. However, some studies showed that other soluble factors involved in the inflammatory response, such as IL-17F, IL-21, IL-22, IL-26, and the chemokines CXCL8 and CCL20, were also produced [33, 76], and they were not studied in our work, being this an important limitation. Therefore, it is important in the future to determine the participation of these factors in the development of tissue damage in our model, principally IL-17F, which shares functions with IL-17A.

Research in human TB is limited due to the difficulty to get samples of the injured lung tissue, and the immune response, pathogenic characteristics and severity is related to the clinical phase of this chronic disease that is also affected by the therapy and social conditions of the patients [95, 96]. Thus, diverse animal models have been created and they substantially contributed to the understanding of the pathogenesis, disease progression and preclinical evaluation of new therapies and vaccines [23]. Relevant models in non-human primates, guinea pigs, rabbits, ruminants, and rodents, among others have been established [17, 22, 23, 97, 98]. Each one of these models has its own attributes that have permitted the study of diverse aspects of TB immunopathogenesis. The rabbit model has been relevant to study the immunopathogenesis produced by *M*. *tuberculosis* or *M*. *bovis* infection because it shows similar characteristics to human TB disease, such as pulmonary necrosis with cavitation [99–101], which was associated with delayed and suboptimal macrophage activation, delayed differentiation, and accumulation of antigen-specific T cells with upregulated networks of IL-4 and B-cell activation [98]. Non-human primates and guinea pig TB models produce similar necrotic lesions than humans, so they are better than conventional strains of mouse models whose granulomas do not develop the necrotic caseous center that is the primary characteristic of human TB [102]. However, we consider our murine model an approach to studying the mechanisms that produce tissue necrosis caused by a highly virulent *M*. *bovis* strain in pneumonic areas not in granulomas, which is the principal limitation of this model. Thus, further research is necessary to establish the most appropriate model for the study of the immunopathology related to necrosis induction in TB pulmonary lesions.

## Conclusion

In conclusion, IL-17A played a protective role in the immune response against high- and low-virulence *M*. *bovis* strain infections in our murine model of progressive pulmonary TB. IL-17A neutralization had a direct effect on increasing the pulmonary bacillary load, as well as on the reduction of cell recruitment for the formation of granulomas, and no evidence was found of IL-17A involvement in lung necrosis. Therefore, further research is needed to dissect the mechanisms related to the development of pulmonary necrosis in TB, with BALB/c mice infected with hypervirulent *M*. *bovis* strain 04–303 being a potentially useful model.

## Supporting information

S1 File Survival *M*. *bovis* infection. DOI: 10.6084/m9.figshare.25549399.
(PZFX)

S2 File CFUs, granuloma, pneumonia and necrosis area in lungs infected with *M*. *bovis* strains. DOI: 10.6084/m9.figshare.25549399.
(XLSX)

S3 File IL-17A total intensity expression in lungs infected with *M*. *bovis* strains assessed by digital pathology. DOI: 10.6084/m9.figshare.25549399.
(XLSX)

S4 File Survival rates of infected with *M*. *bovis* strains and treated mice with IL-17A neutralizing antibodies. DOI: 10.6084/m9.figshare.25549537.
(PZFX)

S5 File CFUs and necrosis area in lungs infected with *M*. *bovis* strains and treated with IL-17A neutralizing antibodies. DOI: 10.6084/m9.figshare.25549537.
(XLSX)

S1 Table Antibodies used for flow cytometry. DOI: 10.6084/m9.figshare.25545913.
(DOCX)
