# Supplementary material for: Effect of IL-17A on the immune response to pulmonary tuberculosis induced by high- and low-virulence strains of Mycobacterium bovis
Source: PLoS One. 2024 Jul 18;19(7):e0307307. doi: 10.1371/journal.pone.0307307 (PMC11257284; doi:10.1371/journal.pone.0307307)
Supplement: S1 Table — DOI: 10.6084/m9.figshare.25545913. (DOCX) [file pone.0307307.s006.docx]

**S6 Table: Antibodies used for flow cytometry.**

| **Antibody** | **Fluorophore** | **Company** |
| --- | --- | --- |
| Ghost-Dye | UV450 | Tonbo; 13-0868 |
| CD3 | redFluor 710 | Tonbo; 800032 |
| CD4 | BV510 | Biolegend; 100559 |
| CD8 | BV711 | Biolegend; 100759 |
| CD11b | FITC | eBioscience; 11-0112-82 |
| Ly-6G | V450 | Tonbo; 75-1276 |
| IFN-γ | BV650 | Biolegend; 505839 |
| IL-4 | PE | BD Biosciences; 554389 |
| IL-17A | PECF594 | BD Biosciences; 562542 |
